# High-resolution tomographic volumetric additive manufacturing

**DOI:** 10.1038/s41467-020-14630-4

**Published:** 2020-02-12

**Authors:** Damien Loterie, Paul Delrot, Christophe Moser

**Affiliations:** Laboratory of Applied Photonics Devices, School of Engineering, Ecole Polytechnique Fédérale de Lausanne, CH-1015 Lausanne, Switzerland

**Keywords:** Mechanical engineering, Synthesis and processing, Laser material processing

## Abstract

In tomographic volumetric additive manufacturing, an entire three-dimensional object is simultaneously solidified by irradiating a liquid photopolymer volume from multiple angles with dynamic light patterns. Though tomographic additive manufacturing has the potential to produce complex parts with a higher throughput and a wider range of printable materials than layer-by-layer additive manufacturing, its resolution currently remains limited to 300 µm. Here, we show that a low-étendue illumination system enables the production of high-resolution features. We further demonstrate an integrated feedback system to accurately control the photopolymerization kinetics over the entire build volume and improve the geometric fidelity of the object solidification. Hard and soft centimeter-scale parts are produced in less than 30 seconds with 80 µm positive and 500 µm negative features, thus demonstrating that tomographic additive manufacturing is potentially suitable for the ultrafast fabrication of advanced and functional constructs.

## Abstract (web summary)

Tomographic additive manufacturing produces complex parts with a wide range of printable materials but remains limited in terms of resolution. Here, the authors tune the étendue of the light source and accurately control the photopolymerization kinetics using an integrated feedback system, leading to the fabrication of high resolution features.

## Introduction

In the last decade, additive manufacturing (AM) has found widespread application, from end-use aerospace components^1,2^ to patient-tailored medical devices^3,4^ and bioprinting of tissues and organs^5–7^. Such applications require AM methods to produce accurate parts with a high throughput and high resolution, as well as to offer a wide range of printable materials.

However, the sequential layer-by-layer operation of existing AM techniques hinders their performances. First, throughput is often limited by the material viscosity^6,8,9^, which in turn restricts the range of processable resins, pastes, powders, and filaments. Moreover, the design freedom and productivity of current layerwise fabrication approaches are reduced by the need to support overhanging structures by struts or a printing bed. The removal of these supports demands extensive manual post-processing of the parts. Layer-by-layer production may also result in anisotropy^10,11^ of the parts’ physical properties that cannot be easily corrected on most AM processes^12^. To improve quality, adaptive feedback control systems can be used as demonstrated for example in metal-based AM^13–16^.

To overcome the geometric constraints and throughput limitations of layer-by-layer light-based AM techniques, namely digital-light processing (DLP) and stereolithography (SLA), multi-beam AM techniques have been proposed^17–20^. In multi-beam AM, the object is not formed by sequentially curing layers of a photopolymer but rather created in a single step by irradiating a transparent resin bath from multiple angles, which results in the local accumulation of light dose and the consequent simultaneous solidification of specific object voxels.

Though such volumetric part generation potentially yields higher throughput (>10^5^ mm^3^ per hour)^21^ than existing DLP and SLA techniques and allows processing more viscous resins (4–93 Pa s)^19,20,22^ and even thermoreversible gels^21^, the smallest feature size demonstrated by multi-beam AM is currently limited to approximately 300 µm^18^.

As opposed to DLP and SLA, where the polymerization extent of a layer is controlled by using highly absorbing resins^23^, volumetric AM requires transparent resins, and the spatial and temporal extent of the volumetric photopolymerization process needs to be controlled by light-shaping to achieve high-resolution printing.

Here, we demonstrate high-resolution (80 µm) volumetric production of centimeter-scale acrylic and silicone parts by feedback-enhanced tomographic reconstruction. First, we show that producing high-resolution features requires a low-étendue illumination system. We then combine this optimized projection source with an integrated feedback system to accurately control the photopolymerization kinetics over the entire build volume and reliably create complex and hollow parts in a matter of seconds (<30 s).

## Results

### Tomographic additive manufacturing

Tomographic AM is based on the simultaneous irradiation of an entire volume of photosensitive resin. The absorption length of the resin is therefore tuned so that the illumination light reaches deeply into the build volume. Therefore, tomographic AM radically differs from layer-by-layer AM where only thin slices of a highly absorbing resin are sequentially cured so that the already processed layers are not overcured by the subsequent slice exposure.

**Fig. 1:**
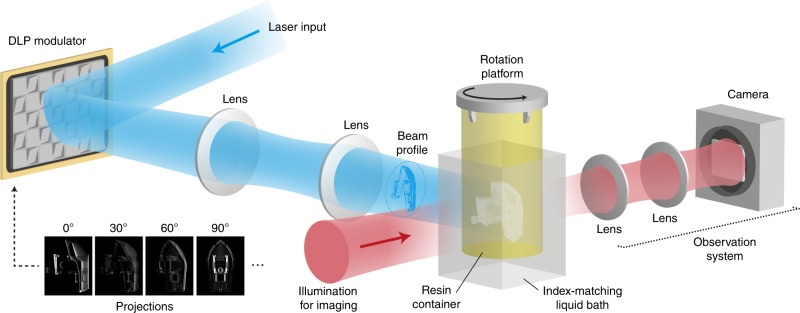
Experimental setup for high-resolution tomographic printing.

The tomographic process is illustrated in Fig. 1. A cylindrical container of resin is set into rotation while it is being irradiated from the side with computed patterns of light. The light patterns are produced by a DLP modulator and they are displayed in synchronization with the rotational movement of the resin container. The patterns represent projections of the object to fabricate as seen from different rotational angles, and they are computed by a Radon transform similarly to X-ray computed tomography^24^.

At any given time, the intensity of a single light pattern is insufficient to solidify the resin completely. However, after the container has been illuminated from every angle by all the light patterns, a three-dimensional distribution of accumulated light dose is created. This three-dimensional dose distribution causes the resin to locally reach its gelation threshold^25,26^, thus resulting in the solidification of the desired object.

The resolution of the printed part and its fidelity to the digital model depends on the interplay of several physico-chemical parameters: the resin’s viscosity and reactivity, the étendue of the illumination patterns and the accuracy of the tomographic dose reconstruction.

### Sedimentation and diffusion of radical species

Counteracting the sedimentation of the object being printed requires using high-viscosity resins, which in turn benefits to the printing resolution since diffusion blurring of the dose distribution is reduced.

The photopolymerization of liquid pre-polymer formulations into solids typically induces shrinkage of 10–15%^27^. In tomographic AM, the resulting increase in density Δ*ρ* of the part being solidified can lead to its sedimentation into the resin bath. For spherical volumes in a liquid of viscosity *μ*, a scaling law for the sedimentation speed *v* is $v \propto \rho /\mu$.

By selecting resins with *µ* > 10 Pa s, no significant sedimentation could be measured over the manufacturing time (20 s) of different relevant geometries, thus showing that sedimentation has a negligible impact on the printing resolution in our current setup (see Supplementary Note 1 and Supplementary Movies 1–2).

Similarly, the diffusion of reactive species, such as oxygen and radicals, is reduced by using highly viscous resins. Oxygen inhibits polymerization by scavenging free radicals or quenching the photoexcited initiator. This inhibiting effect is exploited in continuous liquid interface production^28^ but is detrimental to the object fabrication in DLP and SLA^29^ as it reduces polymer conversion. In tomographic AM, the local depletion of oxygen created by the dose deposition could result in a gradient-induced oxygen diffusion and a potential blurring of the dose distribution over the manufacturing time. However, in a 10 Pa s viscous resin, the diffusion coefficient of oxygen is 1.2 × 10^‒13^ m^2^ s^‒1^, the diffusion of oxygen radical scavengers is thus less than 2 μm over the 20 s manufacturing time and the diffusion blurring of the dose distribution can be ruled out.

### Étendue-limited printing resolution

The printing resolution of tomographic AM is also defined by the étendue of the light source used to produce the illumination patterns.

**Fig. 2:**
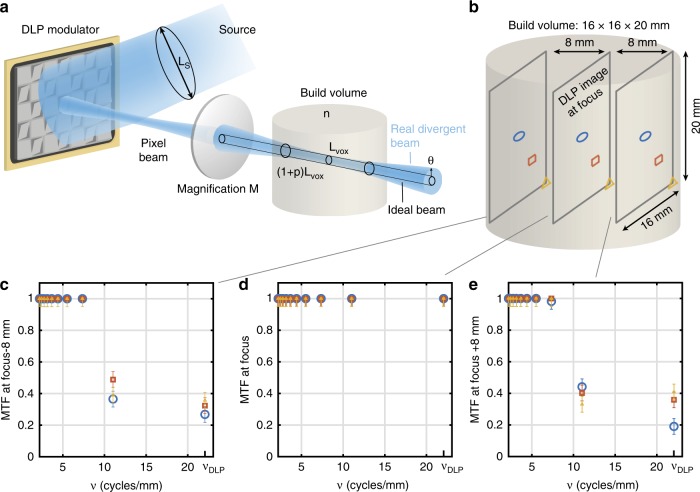
Optical resolution in tomographic AM. **a** étendue-limited optical resolution. **b** Experimental measurement points on-axis (blue circle), at mid-field, (red square) and edge of field (yellow triangle) of the modulation transfer function (MTF) in the build volume of our tomographic printer. **c** Experimental MTF as a function of the spatial frequency 8 mm ahead of the focal plane, **d** at focus and **e** 8 mm after focus. The error bars represent the standard deviation of five repeated measurements at a point at the edge-of-field point at focus, and the error value for the other points were assumed to be the same (see Supplementary Note 3).

Optically, the voxel resolution *L*_vox_ in the center of the build volume is determined by the pixel size of the modulator *L*_p_ and the magnification *M* of the lens system (see Fig. 2). Away from the center of the build volume, the effective pixel size increases proportionally to the divergence of the illumination beam. If we wish to limit the fraction *p* of overlap between pixels at the edge of the build region, then a ray-optics analysis of the projection system leads to the condition that *L*_S_NA_S_ = *npL*_vox_, where *n* is the refractive index of the resin, *L*_S_ is the spatial extent of the illumination source, and NA_S_ is the numerical aperture of the source (the derivation of this formula is available as Supplementary Note 2). In other words, maintaining a high resolution both at the center as well as on the edge of the build volume requires an illumination source with a low étendue *L*_S_ NA_S_.

To achieve high-resolution tomographic AM, six laser diodes were coupled in a square core fiber (*L*_S_ = 70 μm) of numerical aperture NA_S_ = 0.22, yielding a theoretical optical resolution of 23 µm in the center of the 16 mm × 16 mm × 20 mm build volume and of 33 µm on the edge (see Supplementary Note 2 for a detailed calculation). These spread values of the pixel images correspond to maximum spatial frequencies of 21.9 cycles per mm and 15.2 cycles per mm, respectively. The experimental measurements of the modulation transfer function (MTF) performed over the build volume of our tomographic volumetric printer (see Fig. 2c–e and Supplementary Note 3) are consistent with the above theoretical calculations as the maximal spatial frequency of the DLP modulator *υ*_DLP_ = 21.9 cycles per mm can be reached over the whole DLP field in the center of the build volume whereas the printer’s MTF decreases on the edge of the build volume to ~0.4 for a spatial frequency of 11 cycles per mm, which corresponds to a 27 µm optical resolution.

Interestingly, the theoretical cut-off spatial frequency of the optics relaying the DLP image to the build volume is 2NA/*λ* that is to say a 422 cycles per mm spatial frequency and a 1.2 µm optical resolution in our system. Hence, our experimental measurements show that the optical resolution in tomographic volumetric AM is étendue-limited rather than diffraction-limited and that optimizing the étendue of the source allows optimally exploiting the spatial frequencies achievable by the DLP modulator over the printer’s build volume.

### High-resolution printing

**Fig. 3:**
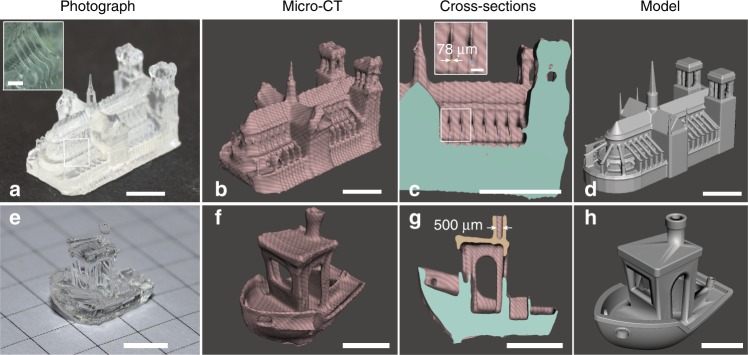
Printed parts and comparison to model. **a** Photograph, **b** micro-CT rendering, **c** micro-CT cross-section, and **d** original model for Notre-Dame. A video recording of the printing of Notre Dame is available as Supplementary Movie 3. **e** Photograph, **f** micro-CT rendering, **g** micro-CT cross-section, and **h** original model for 3DBenchy. Scale bars: 5 mm. In the inset of **a** the scale bar is 1 mm. In the inset of **c** the scale bar is 0.5 mm.

The understanding of the physico-chemical parameters affecting the performances of tomographic AM enabled us to demonstrate high-resolution features in printing time as short as 20 s, as shown in Fig. 3.

Suspended structures as thin as the 80 μm of the arched buttresses of the Notre Dame model were achieved in 19.5 s with an acrylic resin (see Fig. 3a–c and Supplementary Movie 3). Similarly, hollow structures as small as the 500 μm aperture of the chimney of the 3DBenchy model were produced in 25 s in acrylic (see Fig. 3e, f and the micro-CT cross-section in Fig. 3g). Hollow pulmonary artery models made of soft silicone were also produced through tomographic 3D printing and used as a pre-operative training model for vasculature-stitching (see Supplementary Note 5).

As shown in Fig. 3a–c, some sharp corners such as the Notre Dame’s towers were not printed correctly, which is currently a limitation of the dose deposition algorithm as described in the previous section.

### Feedback

To further improve the fidelity of the printed parts to the digital model, we take advantage of the transparency of the build volume to integrate a closed-loop control system.

**Fig. 4:**
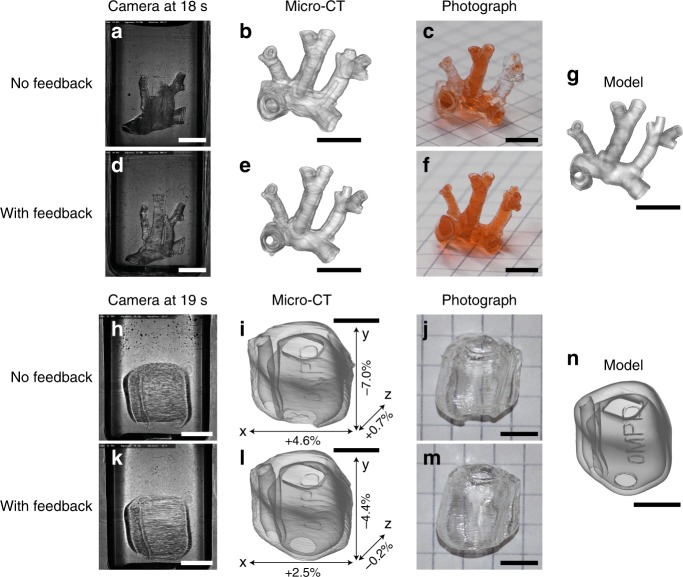
Improving printing accuracy with an integrated feedback system. **a** Video snapshot during the printing of the artery without feedback. **b** Micro-CT scan of the printed artery (without feedback), rendered with transparency to show the occlusions. **c** Photograph of the printed artery (without feedback) perfused with a red dye, to visualize the open channels. **d**–**f** Corresponding data for the artery printed with feedback. **g** Digital model of the printed artery. A comparative video recording of the artery prints is available as Supplementary Movie 4. **h** Video snapshot, (**i**) micro-CT rendering, and **j** photograph of the hearing aid model without feedback. **k**–**m** Corresponding data for the hearing aid with feedback. **n** Hearing aid model. **i** and **l** show the deviation of the printed parts with respect to the digital model **n**, as measured by the micro-CT. The scale bars are 5 mm.

As shown in Fig. 1, a camera observes the build volume at a 90° angle relative to the direction of illumination. This camera records images continuously during the build procedure, showing which parts of the model appear at which time point. In turn, this information can be used as feedback to stop illuminating parts that are already solid or boost the dose in places which solidify more slowly. While this correction could in principle be done in real time during a print, on-the-fly pattern correction is not possible on our DMD module and would instead require dedicated electronics or an FPGA. Instead, we demonstrate the correction sequentially in this paper (that is to say the data from one print is used to correct subsequent prints). The results are shown in Fig. 4. First, a model of the pulmonary artery of a mouse (Fig. 4g) was printed without feedback in 19 s. The central blood vessel (Y-branch) appeared with a delay compared to the rest of the structure (Fig. 4a). After exposing for 19 s, the Y-branch appeared but the other vessels were clogged, as shown in the micro-CT scan of the printed object (Fig. 4b) and the photograph in Fig. 4c. Using the recording from the first print, a second print was made with the same exposure time and a spatial adjustment of the dose. With this correction, the various parts of the object solidified simultaneously (see Fig. 4d and Supplementary Movie 4) which led to a higher geometric fidelity (Fig. 4e), where the branches are visible without being obstructed (Fig. 4f). This result could not be obtained simply by lowering the exposure time without feedback, since the Y-branch is then not printed, as discussed in Supplementary Note 4. Similarly, a model of a shell for a hearing aid was printed in 21.6 s without feedback, leading to a delayed appearance of the bottom of the part (in Fig. 4h) and defects on the printed parts (Fig. 4i, j). With feedback (Fig. 4k–m), the openings of the hearing aid shell are fully defined and the overall object dimensions correspond more accurately to the model of Fig. 4n.

In summary, we showed that both the étendue of the illumination system and the resin viscosity are critical parameters to produce high-resolution features with tomographic AM. We also demonstrate that the transparency in tomographic AM can be exploited to implement a feedback system to accurately control the photopolymerization kinetics. The 80 µm positive and 500 µm negative feature sizes achieved over centimeter-scale parts pave the way for ultrafast production of precise constructs such as functional tissue or organ models^21,30,31^.

## Methods

### Optical setup

Six 405 nm laser diodes, with a combined nominal power of 6.4 W, are collimated and combined into a single beam with closely spaced mirrors. The combined beam is then coupled into a square-core optical fiber (CeramOptec WF 70 × 70/115/200/400 N, core size 70 μm by 70 μm, numerical aperture 0.22), which allows homogenizing the beam from the laser diodes and matching to the rectangular aperture of the modulator for increasing the light efficiency. The output of the fiber is then magnified and projected onto a digital micromirror device (DMD, Vialux V-7000) via an aspheric lens (Thorlabs C151TMD-A *f* = 2 mm) and a set of orthogonal cylindrical lenses (Thorlabs LJ 1267L1 f = 250 mm, Thorlabs LJ1996L1 f = 300 mm). The cylindrical lenses have different focal lengths, which allows adjusting the square beam from the fiber to the rectangular area of the DMD.

The DMD in our setup is fixed on a rotational mount in such a way that the rotation axis of the device corresponds to the diagonal tilt axis of the micromirrors. The rotation angle was approximately 7° to account for the blazed grating effect introduced by the matrix of micro-mirrors.

The surface of the DMD is imaged via a 4f-system (Thorlabs AC254-100-A-ML *f* = 100 mm and Edmund Optics #67-222 f = 250 mm) into a cylindrical glass vial containing the photopolymer. In the Fourier plane, an aperture blocks the unwanted diffraction orders from the DMD. In this setup, the addressable volume inside the photopolymer is approximately 17.5 mm × 17.5 mm × 23 mm. When all the DMD pixels are in their “ON” state, the power of the light beam sent into the vial is approximately 1.6 W.

### Projection algorithm

The light patterns were calculated using a filtered back-projection algorithm. First, 3D models in STL format were converted to a three-dimensional voxel map, that is to say a 3D matrix where the values “1” indicate the presence of the object and “0” its absence at each particular location in space. For each 2D section of this matrix, projections were calculated over a 360° grid of angles with a spacing of 0.6° (see also Supplementary Note 2), using the “radon” function in MATLAB. This function numerically calculates the Radon transform:

$${\cal{R}}f\left( {t,\theta } \right) = \mathop {\smallint }\limits_{ - \infty }^{ + \infty } f\left( {q\sin \theta + t\cos \theta ;\, - q\cos \theta + t\sin \theta } \right)\,{\rm{d}}q.$$

Here, *θ* represents the projection angle, *q* is the spatial coordinate over which the object is integrated (direction of the projection), and *t* is the remaining spatial coordinate (shift at which each line integral is calculated). The projections were subsequently filtered with a Ram–Lak filter in the Fourier domain. This filter yields a set of projections which, when projected back into an empty volume, result in theory in a perfect reconstruction of the object^24^. Therefore, these are the light patterns that would need to be physically projected into the build volume at their respective angles. In practice, the Ram–Lak filter produces projections with both positive and negative values, the latter of which cannot physically be projected into the volume. For this, a simple threshold was used to set the negative values to 0. While this makes the resulting reconstruction approximate instead of exact, we note that most of the light dose is still concentrated in the shape of the object. The gelation threshold of the photopolymer then makes that these are the only parts of the volume that solidify. While other parts of the resin do receive a certain amount of unwanted light dose, this is not enough to cross the gelation threshold in the majority of cases.

### Feedback algorithm

For the feedback correction, a camera recorded intensity images of the build volume in synchronization with the rotation. The build volume was illuminated from the back (transmission imaging) by an expanded and collimated laser beam at 671 nm. The images were filtered and down-sampled to reduce noise and speed up processing respectively. As seen in Fig. 4(a, d, h, k), the resin appears darker when it solidifies (due to refraction and scattering). We first recorded a set of reference images of the rotating build volume before starting the actual exposure. We then calculated the difference between each subsequent turn (during the exposure) with this reference set. A simple threshold was then used to detect which parts of the volume had become solid at which time. Spatially, this information is still two dimensional at this point because it is derived from 2D transmission images of the entire build volume. In order to build a three-dimensional map of the time needed for solidification, we ‘back-projected’ the time values into a 3D grid. When a transmission image at a particular angle and time showed ‘solid pixels’ (as measured by the thresholding procedure), the time of solidification was recorded in the 3D volume in a line corresponding to these pixels and oriented along the direction of projection. If a later transmission image from any other angle showed no solidification or a transition to solid state at a later time for the same group of pixels in 3D, then the recorded solidification time was increased for those pixels. The resulting 3D volume of ‘solidification times’ directly gives the required intensity correction for the next print. Indeed, the dose *D* is related to intensity *I* and time *t* as *D* = *It*. If a given part of the object takes a longer time *t* to solidify, the intensity *I* in this part simply needs to be increased proportionally to make it solid at the same time as the rest of the model. After this 3D intensity adjustment, corrected Radon projections can be calculated from the corrected model of the object using the projection algorithm. Further details on the feedback algorithm can be found in Supplementary Note 6.

### Resin

The photosensitive resin used in this work was prepared by magnetically stirring at 500 rpm di-pentaerythritol pentaacrylate (SR399; Sartomer, France) with 0.6 mM phenylbis(2,4,6-trimethylbenzoyl)phosphine oxide (97%; Sigma Aldrich, USA) in glass jars heated at 100 °C for 1 h. To remove bubbles trapped in the resin, the vials were also centrifuged at 500 rpm for 30 s prior to use.

### MicroCT measurements

The 3D benchy and Notre-Dame parts were scanned using a SkyScan 1076 (Bruker) with a 9 µm pixel resolution. The CT-scans of the artery and hearing aid models were obtained on an industrial-grade EasyTom S scanner (Rx Solutions, France) and reconstructed using X-Act software (Rx Solutions). The hearing aid scans were registered to the model using VGStudio (Volume Graphics) and the dimensional errors reported in Fig. 4 were measured at the same location on both models along three orthogonal axes using myVGL (Volume Graphics).

## Supplementary information

Supplementary Information

Description of Additional Supplementary Files

Supplementary Movie 1

Supplementary Movie 2

Supplementary Movie 3

Supplementary Movie 4

## Data Availability

The data that support the findings of this study are available from the corresponding author upon reasonable request.

## Code availability

The computer code for the feedback correction is available from the corresponding author upon reasonable request.
