# Supplementary material for: High-resolution tomographic volumetric additive manufacturing
Source: Nat Commun. 2020 Feb 12;11:852. doi: 10.1038/s41467-020-14630-4 (PMC7015946; doi:10.1038/s41467-020-14630-4)
Supplement: Supplementary file 1 — Supplementary Information [file 41467_2020_14630_MOESM1_ESM.pdf]

SUPPLEMENTARY MATERIALS

High-resolution tomographic volumetric additive manufacturing

Damien Loterie[†,1], Paul Delrot[†,1,*] and Christophe Moser[1]

[1] Laboratory of Applied Photonics Devices, School of Engineering,
Ecole Polytechnique Fédérale de Lausanne, CH-1015, Lausanne, Switzerland

[†] These authors contributed equally
* corresponding author: paul.delrot@epfl.ch

Supplementary Note 1: Sedimentation of the printed part

In volumetric AM, the photopolymerization process is stopped at gelation threshold, when the polymer conversion is $\kappa = \frac{1}{\alpha - 1}$ where α is the number of possible bonds of a monomer molecule [1]. For the penta-acrylate resin used in this work, the corresponding polymer conversion at gelation is $\kappa = 11\%$. At this polymer conversion, we measured a limited shrinkage of our multifunctional resin 1.4% ± 0.1% compared to typical resin shrinkage of 10 to 15% [2]. This limited shrinkage of our resin further accounts for a negligible sedimentation rate. Hence, no motion could be observed over a 20 second time span after the printing process was completed on spheres of diameters between 500 µm and 4 mm (see Supplementary Movie 1). The motion measurement was performed with a camera having a pixel pitch equivalent to 26 µm in the build volume.

As the shape of objects affects their drag coefficient and consequently their sedimentation rate, the motion of an object with a streamlined shape (see Supplementary Figure 1a), more prone to sedimentation than spheres, was measured over time during and after the printing

process. As shown in Supplementary Figure 1b and Supplementary Movie 2, no significant motion of the object could be measured over the tomographic exposure process. The settling time of the streamlined body motion is about 20 seconds. Hence, the streamlined object exhibits a significant motion only 15 seconds after the printing process is completed, which shows that the effect of sedimentation on the printing resolution is negligible in our current tomographic additive manufacturing system.

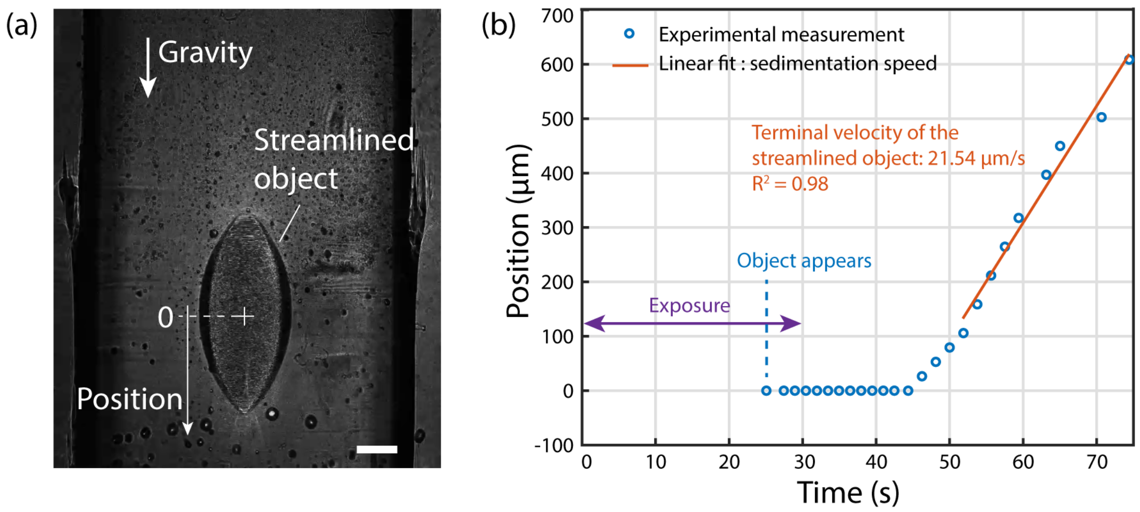

Supplementary Figure 1: Effect of sedimentation on the printed parts. (a) Experimental setup for sedimentation rate measurement, scale bar: 2mm. (b) Motion of the streamlined object over time, the error margin on the position measurement is 26µm due to the limited optical resolution of the camera.

Supplementary Note 2: Theoretical optical resolution

The two main factors that determine the optical resolution in tomographic additive manufacturing are the resolution of the projections and the étendue of the source.

The resolution of the projections itself has two components: one is the effective pixel size in the build volume, and the other is the angular step size at which the projections are calculated. The former determines the spatial resolution near the center of the build volume, and the latter imposes an additional limit of resolution at the edge of the build volume due to angular sampling. At the edge of a build volume with a diameter *d*, if the number of

projections per full turn is N, the perimeter π d is divided in N intervals leading to a resolution of at most π d / N. For example, in our experiments we projected 600 frames per full turn (0.6° spacing). At a radius of r = 7.5mm away from the center of the build volume (which corresponds approximately to the size of the Notre Dame model), the perimeter of 2 π r = 47.1 mm is divided in 600 intervals, leading to a sampling period of 79 µm at the edge of the build volume. Note that halfway between the center and the edge of the volume, the resolution limit due to angular sampling is just 39 µm. Incidentally, we note that when a very high number of angular steps is desired, the maximum display rate of the DMD projector (290 Hz in this work) puts an upper limit on the rotation speed of the build volume. This has not been an issue in this work, but could be limiting in certain applications. Finally, we note that an MTF analysis that models the angular resolution limit in this process as motion blur can be found in the supplementary materials of Kelly et al.[3].

The second main factor influencing the resolution is the étendue of the source. With a resin of refractive index n = 1.47 and a DMD pixel size of 23 µm after magnification, the divergence of the light beam causes a maximum spread of approximately p = 46%, which results in DMD pixel size of 33 µm on the edge of the build volume.

The formula $L_S \, NA_S = n \, p \, L_{vox}$ is derived as follows based on ray optics considerations. We assume that we use a square-shaped source of light with a side-length of L_S and a numerical aperture of NA_S. This source is magnified by an ideal 4f lens system to fit the active area of a DMD, which we assume to be a square with side-length L_{DMD}, a pixel size of L_{pixel} and a number of pixels (in one dimension) of $N_{pixel} = L_{DMD} / L_{pixel}$. The magnification from the source to the DMD is $M_1 = L_{DMD} / L_S$. After being modulated by the DMD, the light pattern is relayed by a second ideal 4f lens system so that it fits the size of the build volume, which is a cylinder of diameter L_{volume} and the index of refraction is n. The second magnification factor is

$M_2 = L_{\mathrm{volume}} / L_{\mathrm{DMD}}$. The effective voxel side-length in the center of the build volume will be therefore be $L_{\mathrm{vox}} = M_2\, L_{\mathrm{pixel}}$. At the front and back of the build volume, which are at a distance $L_{\mathrm{volume}} / 2$ from the center, the effective voxel size will be larger due to the divergence of the light beam. Assuming the light from each DMD pixel has an effective numerical aperture of $\mathrm{NA}_{\mathrm{volume}}$ inside the build volume, then the divergence angle is given by $n \sin\phi = \mathrm{NA}_{\mathrm{volume}}$. Over a distance $L_{\mathrm{volume}} / 2$, this translates to an increase of $\tan\phi\, L_{\mathrm{volume}}$ of the voxel size, or $L_{\mathrm{volume}}\, \mathrm{NA}_{\mathrm{volume}} / n$ with a small-angle approximation. If we wish to limit the relative increase of the voxel size at the edge of the volume to a factor p, we have: $L_{\mathrm{volume}}\, \mathrm{NA}_{\mathrm{volume}} / n = p\, L_{\mathrm{vox}}$. Now, since L_{volume} is related to the source size by $L_{\mathrm{volume}} = M_1\, M_2\, L_S$ and $\mathrm{NA}_{\mathrm{volume}}$ is related to the source numerical aperture by $\mathrm{NA}_{\mathrm{volume}} = \mathrm{NA}_S / (M_1\, M_2)$, we finally obtain $L_S\, \mathrm{NA}_S = n\, p\, L_{\mathrm{vox}}$, a simple design rule relating the achievable resolution to the source parameters.

Supplementary Note 3: Modulation transfer function measurements

The modulation transfer function (MTF) of an optical system is defined as the capability of the optical system to transmit spatial frequencies from the object plane, the DLP in our tomographic volumetric printer, to the image plane, the build volume in our printer

The MTF at a given spatial frequency ν is measured or computed as the contrast C of sinusoidal grating of the specified spatial frequency ν through the optical system,

$$C = \frac{I_{\mathrm{max}} - I_{\mathrm{min}}}{I_{\mathrm{max}} + I_{\mathrm{min}}}$$

where I_{max} and I_{min} are respectively the maximal and minimal intensity values of the image of the grating.

In this work, the MTF of our tomographic volumetric additive manufacturing system was measured by displaying gratings of different spatial frequencies on the DLP modulator that is

to say 4.4 to 21.9 cycles per mm in the image plane, which corresponds to half-periods of 1 to 10 pixels on the DLP modulator.

The images of the DLP-generated gratings were then measured in different planes of the build volume as shown in Figure 2(b-e). The gratings were displayed in the center of the DLP modulator, which corresponds to on-axis measurement, at half the DLP diagonal, 5 mm tangentially and 4 mm sagitally away from the optical axis, which corresponds to mid-field measurement, and at the edge of the DLP diagonal, 8 mm tangentially and 10 mm sagitally away from the optical axis, which correspond to edge of field measurement.

The imaging planes were chosen to demonstrate the étendue-resolution criterion described in this work. In other words, the étendue-limited optical resolution depends on the magnification of the afocal system relaying the DLP image to the build volume [4] (see Supplementary Note 2). In our system, the DLP is magnified to print over a 16 mm x 16 mm x 20 mm build volume (width, length and height). Therefore, measurements were taken in the focal plane (center of the build volume) and ±8 mm apart from the focal plane, which correspond to the edges of the build volume.

The gratings' image were measured by performing high-dynamic range (HDR) imaging with a camera (DMK 23U445, Imaging Source, Germany) having a sufficiently small pixel size (3.75 µm pixel size versus a theoretical Nyquist half-period of 11.4 µm) to avoid aliasing effects. During the measurements, the resin container and the index-matching liquid bath shown in Figure 1 of the main text were removed to allow placing the camera's sensor at the different planes.

One should note that owing to the tilt of the DLP modulator, the image of the DLP is also tilted and the field MTF measurements were performed in the corresponding tilted planes. In our

setup, the measured focal shift between the on-axis optimal focus and the edge-of-field optimal focus is ~3 mm.

The HDR images of the gratings were then fitted to a sinusoid function in Matlab® to extract the minimal and maximal intensity values of the grating images and compute the MTF.

MTF measurements and processing were repeated 5 times for the edge-of-field point at focus in order to estimate the standard deviation on the MTF values. Measuring the errors bars for each data point in this way would be impractically long, therefore the error was assumed to be similar for all MTF values and the same error bar was applied to all the MTF data points.

No significant difference was measured between sagittal and tangential MTFs. Only the sagittal measurements are displayed in Figure 2(c-d).

Finally, one should note that the MTF evolves non-monotonically through focus owing to the divergence of the source that creates overlap of the grating image as it propagates. This effect was modelled by performing with the same physical characteristics (NA = 0.0047 on the DLP, wavelength: 400 nm, afocal system made of lenses Thorlabs LA4874-UV and Edmund Optics #67-222) as our optical system a ray propagation through focus in Matlab® of a grating with a 6 pixel period (see Supplementary Figure 2a). The resulting theoretical through-focus MTF is consistent with the experimental through-focus MTF measurement performed on our printer with the same grating period (see Supplementary Figure 2b). Interestingly, another theoretical result matched by experimental measurement is that the zeros of the through-focus MTF also depends on the spatial frequency of the grating (results not shown here). This is the expected behavior of the modulation transfer function as described in the literature (see W. Smith, "Modern Optical Engineering", p. 394, McGraw Hill Professional, (2007)).

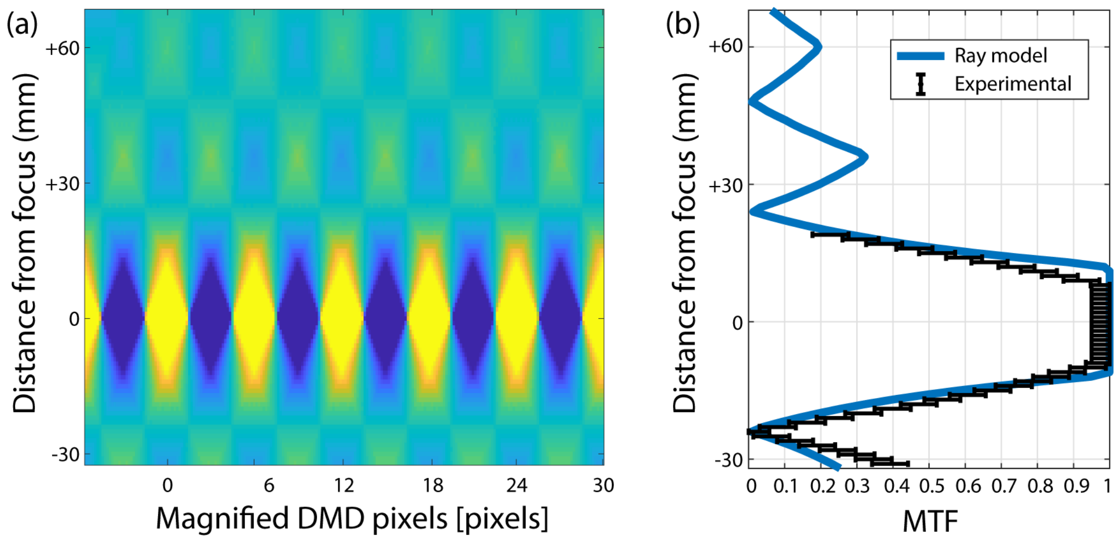

Supplementary Figure 2: Through-focus MTF. (a) Ray simulation of the through-focus propagation of a DMD-generated 6-pixel period grating. (b) Model and experimental through-focus MTF for a 6 pixel DMD period. The error bars represent the standard deviation after repeating the measurement and processing 5 times for a point at focus. The error value for the other points were assumed to be the same (see Supplementary Note 3).

Supplementary Note 4: Effect of changing the exposure time

As discussed in the main text, lowering the exposure time alone (i.e. without using feedback) cannot produce a well-defined print on the artery model. This is evidenced by Supplementary Figure 3. In the artery with feedback (Supplementary Figure 3a), all the vessels are open and well-defined as can be seen in the dye-perfused photograph (Supplementary Figure 3e). In the artery without feedback (Supplementary Figures 3b and f), the central branch is open, but the side-branches are clogged. Progressively lowering the exposure time opens the side-branches at the expense of losing the central branch entirely (Supplementary Figures 3c, d, g and h).

We note that washing away the uncured resin from these artery models requires special care as the resin is viscous and the tubes are very thin. We used the following process on all artery models to ensure consistent results:

- After printing, we separated the part from the unpolymerized resin using a sieve with wide opening. We let the uncured resin flow away during 3 minutes.
- We then immersed the part in warm isopropyl alcohol (IPA) at 45 °C in a standard 50 ml centrifuge tube. The higher temperature was to lower the viscosity of the resin so that it would be better able to flow out of the part.
- We used a vortex mixer (Scientific Industries Vortex Genie 2) on a mild setting (level 3) for 30 s to gently shake the tube with the part in warm IPA.
- We replaced the IPA in the tube with new warm IPA.
- We repeated the vortex and IPA replacement step 5 times in total for each part.

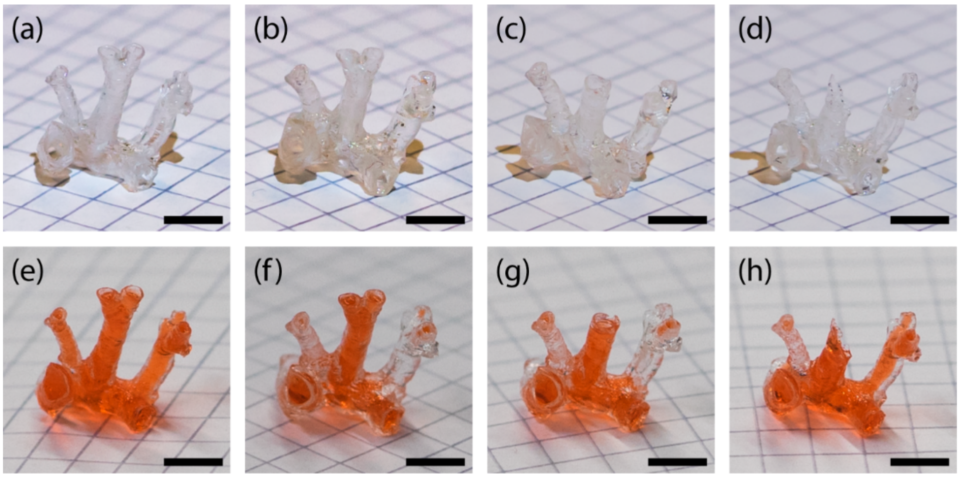

Supplementary Figure 3: Series of prints with different exposure times. (a) Photograph of the artery model printed with feedback with an exposure time of 19 s. (b) Artery printed without feedback during 19 s, (c) 18 s and (d) 17 s. (e-f) Corresponding dye-perfused photographs. Scale bars are 5 mm.

Supplementary Note 5: Volumetric AM of true silicone

Volumetric AM allows using highly-viscous resins with a wide range of mechanical properties. To produce surgical models that mimic the mechanical properties of biological tissues, such

as cartilage[4] or arteries[5], we developed a thiol-ene silicone resin with low elastic moduli of 280 kPa and large elongation at break of 88%.

The developed silicone resin is a mixture of 93 wt% vinyl-terminated PDMS 62 kg mol^{-1} (DMS-V41; Gelest, USA), 4.7 wt% fumed silica reinforced vinyl-terminated PDMS 28 kg/mol (DMS-V31S15; Gelest, USA), 2.3 wt% (mercaptopropyl) methylsiloxane – dimethylsiloxane 3.6 kg mol^{-1} (GPC-367;Genesee Polymer Co., USA) and 2.25 mol m^{-3} ethyl (2,4,6-trimethylbenzoyl) phenylphosphinate (TPO-L, 95%; Fluorochem, UK). This mixture complies with the viscosity and transparency needed for volumetric AM. The scattering properties of the resin were challenging to optimize since we experimentally observed that mixtures with thiol crosslinkers having functionality larger than about 4 to 6 will yield turbid resins, not suited for volumetric AM.

Using this silicone resin, we were able to print hollow vasculature models (see Supplementary Figure 4a) that were stitched by a surgeon (Prof. Dr. Med. Broome). Filling the stitched parts with a stained liquid shows that the parts are correctly sealed (see Supplementary Figure 4b).

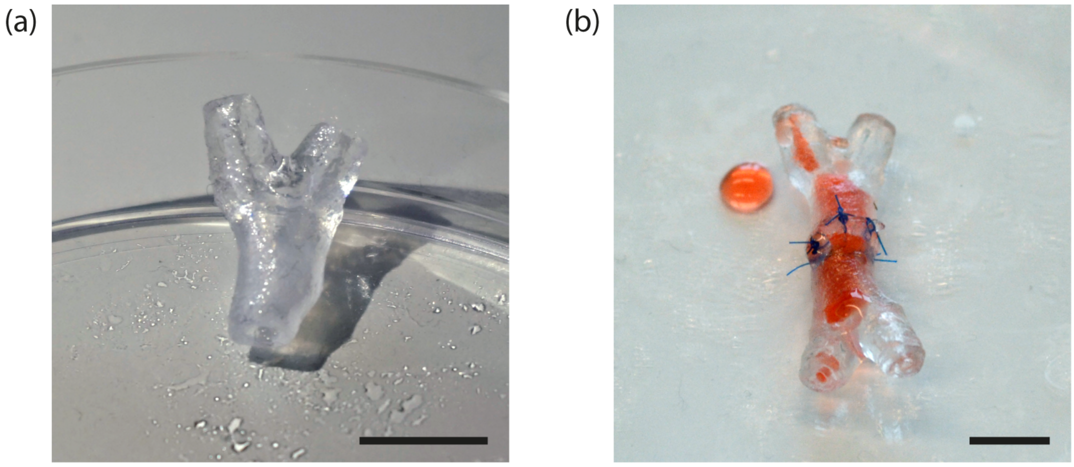

Supplementary Figure 4: Printing of silicone parts. (a) Hollow vasculature model produced in silicone through volumetric AM in 15s. (b) Two vasculature models stitched together, a stained liquid was inserted in the structure to ensure proper sealing. Scale bar: 10 mm.

Supplementary Note 6: Feedback algorithm

In this section, the feedback algorithm is explained in more detail with the help of Supplementary Figure 5. Before the exposure starts, the camera acquires a set of background images at various rotation angles of the vial: $I_{\mathrm{bg}}(x', z', \theta)$. These background images are to be used as reference images after the exposure starts. The axes x' and z' are defined in Supplementary Figure 5 (step 1). During the printing procedure, the vial rotates several times while light patterns are being displayed into it. During each rotation n, the camera records at each angle θ a new image of the vial $I_{\mathrm{rec}}(x', z', \theta, n)$. Each of these images corresponds to a specific time point t in the exposure of the vial, which is uniquely determined by the rotation number and angle: $t = t(\theta, n)$. The relationship between t, θ and n is shown in step 2 of Supplementary Figure 5 for a representative set of images. In step 3, the difference images are calculated between the recordings during exposure and the reference images:

$$I_{\mathrm{diff}}(x', z', \theta, n) = I_{\mathrm{bg}}(x', z', \theta) - I_{\mathrm{rec}}(x', z', \theta, n)$$

In step 4, a threshold T is applied to detect changes compared to the empty vial images $I_{\mathrm{bg}}(x', z', \theta)$:

$$I_{\mathrm{thresh}}(x', z', \theta, n) = \begin{cases} 1 & \text{if } I_{\mathrm{diff}}(x', z', \theta, n) > T \\ 0 & \text{elsewhere} \end{cases}$$

This threshold was chosen here as:

$$T = \frac{1}{3} \max I_{\mathrm{diff}}$$

Based on the thresholded images, a temporal map is constructed in step 5 by looking up for each angle θ and location (x', z') at which time the pixel permanently passed the threshold (i.e. for the current rotation as well as all the subsequent rotations):

$$t_{\mathrm{poly}}(x', z', \theta) = \min_{n} \{ t(n, \theta) \mid \forall\, n' \geq n : I_{\mathrm{thresh}}(x', z', \theta, n') = 1 \}$$

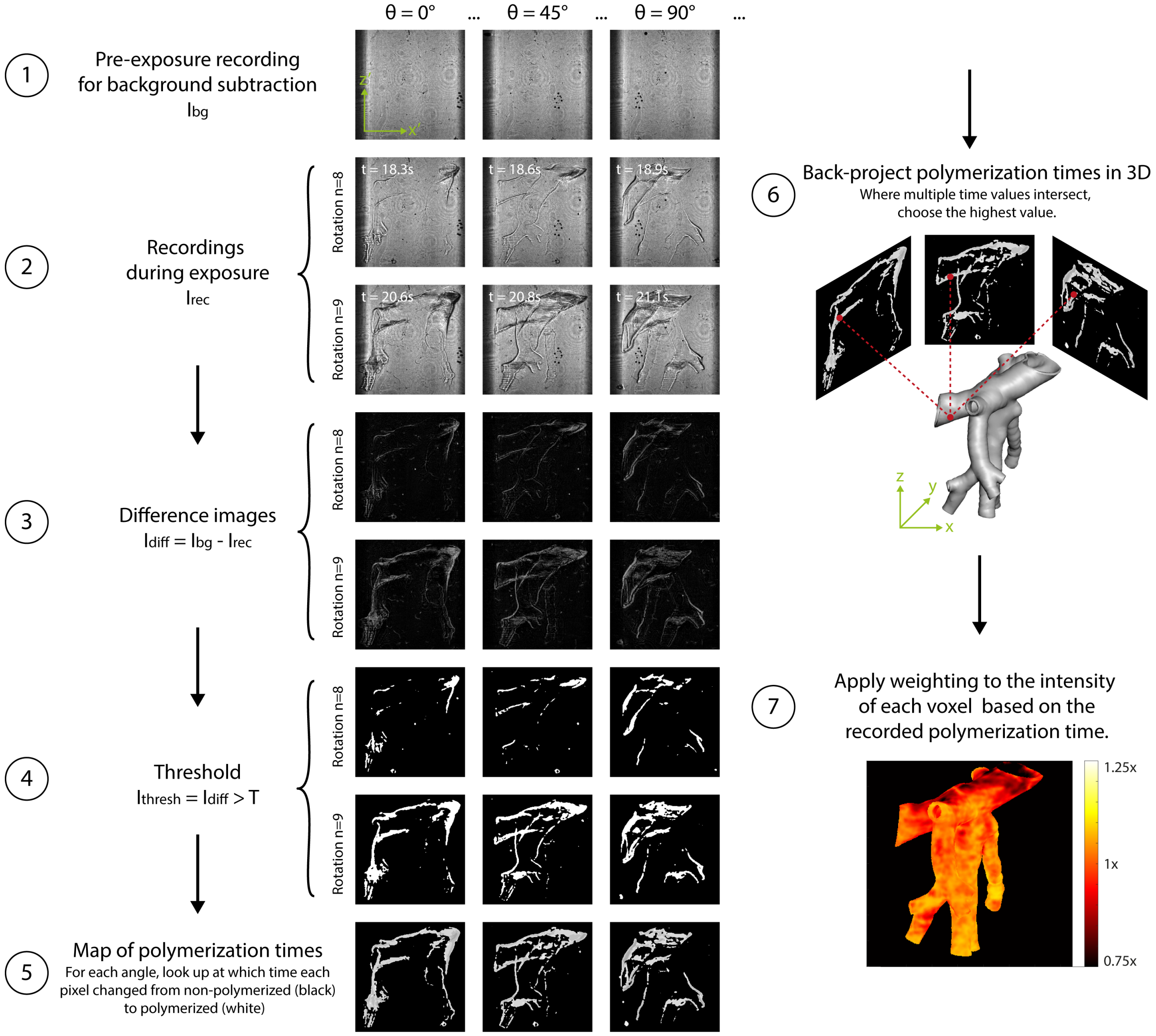

Supplementary Figure 5: Detailed diagram of the data processing for feedback. (1) Background subtraction. (2) Camera recordings. (3) Difference images. (4) Threshold step. (5) Map of polymerization times. (6) Back-projection step. (7) Heat map of the resulting volume intensity correction.

This indicates at what times and position a material transition was detected from each viewpoint θ. Pixels for which no transition is detected are set to infinity. In step 6, this information is back-projected in 3D using the following procedure:

$$t_{3\mathrm{D}}(x,y,z) = \max_{\theta}\left\{t_{\mathrm{poly}}\left(x\cos\theta - y\sin\theta\,, z, \tan^{-1}(y,x)\right)\right\}$$

where $\tan^{-1}(y,x)$ is the 2-argument arctangent function (atan2). This procedure is similar to the inverse Radon transform, except that the combining operator here is the maximum

operator instead of an integral. In other words, for each voxel in 3D space we select the latest time at which it was seen transitioning from below-threshold to above-threshold from any given angle θ. If no data is available for some of the voxels, these are filled in with the nearest neighboring value in 3D. In the final step 7, the dose in each voxel is modulated using the temporal information of step 6: voxels that took longer to transition get a proportionally higher dosage in the next print, while voxels that transitioned faster are proportionally attenuated.

Supplementary References

1. Odian, G. *Principles of Polymerization*. (John Wiley & Sons, Hoboken NJ, 2004).
2. Klang, J. A. Radiation-curable hyperbranched polyester acrylates. *Paint and Coatings Industry* **23,** 98–101 (2007).
3. Kelly, B. E. *et al.* Volumetric additive manufacturing via tomographic reconstruction. *Science* **363,** 1075–1079 (2019).
4. Gross, H. *Handbook of Optical Systems*. (Wiley-VCH Verlag GmbH & Co. KGaA, 2005). doi:10.1002/9783527699223
5. Wallin, T. J. *et al.* Click chemistry stereolithography for soft robots that self-heal. *Journal of Materials Chemistry B* **5,** 6249–6255 (2017).
6. Claes, E. *et al.* Mechanical properties of human coronary arteries. *Conf Proc IEEE Eng Med Biol Soc* **2010,** 3792–3795 (2010).
