# Supplementary material for: High-resolution tomographic volumetric additive manufacturing
Source: Nat Commun. 2020 Feb 12;11:852. doi: 10.1038/s41467-020-14630-4 (PMC7015946; doi:10.1038/s41467-020-14630-4)
Supplement: Supplementary file 2 — Description of Additional Supplementary Files [file 41467_2020_14630_MOESM2_ESM.pdf]

Description of Additional Supplementary Files

File Name: Supplementary Movie 1
Description: Printing of spheres for sedimentation measurements

File Name: Supplementary Movie 2
Description: Printing of a streamlined shape for sedimentation measurements

File Name: Supplementary Movie 3
Description: Printing of the Notre Dame model

File Name: Supplementary Movie 4
Description: Comparison of printing the artery model with and without feedback
